# Component costs of foodborne illness: a scoping review

**DOI:** 10.1186/1471-2458-14-509

**Published:** 2014-05-26

**Authors:** Taylor McLinden, Jan M Sargeant, M Kate Thomas, Andrew Papadopoulos, Aamir Fazil

**Affiliations:** 1 Department of Population Medicine, Ontario Veterinary College, University of Guelph, Guelph, Ontario, Canada; 2 Centre for Public Health and Zoonoses, University of Guelph, Guelph, Ontario, Canada; 3 Centre for Food-borne, Environmental and Zoonotic Infectious Diseases, Public Health Agency of Canada, Guelph, Ontario, Canada; 4 Laboratory for Foodborne Zoonoses, Public Health Agency of Canada, Guelph, Ontario, Canada

**Keywords:** Scoping review, Cost-of-illness, Foodborne illness, Component costs, Knowledge synthesis

## Abstract

**Background:**

Governments require high-quality scientific evidence to prioritize resource allocation and the cost-of-illness (COI) methodology is one technique used to estimate the economic burden of a disease. However, variable cost inventories make it difficult to interpret and compare costs across multiple studies.

**Methods:**

A scoping review was conducted to identify the component costs and the respective data sources used for estimating the cost of foodborne illnesses in a population. This review was accomplished by: (1) identifying the research question and relevant literature, (2) selecting the literature, (3) charting, collating, and summarizing the results. All pertinent data were extracted at the level of detail reported in a study, and the component cost and source data were subsequently grouped into themes.

**Results:**

Eighty-four studies were identified that described the cost of foodborne illness in humans. Most studies (80%) were published in the last two decades (1992–2012) in North America and Europe. The 10 most frequently estimated costs were due to illnesses caused by bacterial foodborne pathogens, with non-typhoidal *Salmonella* spp. being the most commonly studied. Forty studies described both individual (direct and indirect) and societal level costs. The direct individual level component costs most often included were hospital services, physician personnel, and drug costs. The most commonly reported indirect individual level component cost was productivity losses due to sick leave from work. Prior estimates published in the literature were the most commonly used source of component cost data. Data sources were not provided or specifically linked to component costs in several studies.

**Conclusions:**

The results illustrated a highly variable depth and breadth of individual and societal level component costs, and a wide range of data sources being used. This scoping review can be used as evidence that there is a lack of standardization in cost inventories in the cost of foodborne illness literature, and to promote greater transparency and detail of data source reporting. By conforming to a more standardized cost inventory, and by reporting data sources in more detail, there will be an increase in cost of foodborne illness research that can be interpreted and compared in a meaningful way.

## Background

Foodborne illnesses are an important public health problem worldwide [1]. The World Health Organization (WHO) has created an initiative to estimate the global burden of foodborne illnesses, and they have stated that the achievement of certain Millennium Development Goals are being directly compromised due to foodborne illness [2,3]. However, governments have finite resources with which to address the health of their populations, and thus require high-quality scientific evidence to prioritize resource allocation. Accurate burden of illness estimates are useful for decision makers seeking to allocate resources to address the issues caused by foodborne pathogens [4,5]. The cost-of-illness (COI) methodology is one technique used to estimate the economic burden of a disease [6]. However, there is concern in the scientific literature that COI estimates are limited in usefulness, due to variability in their execution (e.g., varying cost inventories and study methodologies), and a lack of transparency and detail when describing such methodologies. Of particular concern are the differing cost inventories being used when estimating the cost of foodborne illnesses [7-10].

The COI approach traces the flow of resources associated with adverse health outcomes through the quantification of measurable individual and societal level costs [7,11,12]. Costs at the individual level are divided into direct and indirect costs. Direct costs represent the value of goods, services, and other resources consumed in providing care due to an illness [13]. These include medical care expenditures associated with the diagnosis, treatment, and management of a disease in an individual. Indirect costs represent productivity losses due to illness or death and intangible costs such as pain and suffering. Costs associated with overhead activities that are shared amongst individuals and expenditures incurred in the process of seeking care are also indirect costs. Operational expenditures for healthcare facilities and personal transportation costs are examples of indirect costs [14]. Costs incurred at the population level are deemed societal costs [8,9,12], which are costs that cannot be completely attributed to an individual’s illness but can be incurred when a person or a group of people become ill [15]. Societal costs primarily include expenditures incurred by industry and government [16]. Component costs are the specific costs that make up the above categories, and all of the costs included in a COI estimate comprise the cost inventory for that particular study [17].

Many studies employing a COI methodology have demonstrated that foodborne illnesses generate a considerable disease burden and economic loss [11]. According to the United States Department of Agriculture (USDA), foodborne illness costs the United States economy between $10-83 billion United States dollars (USD) per year [18]. In Australia and New Zealand, the cost of foodborne illness has been estimated at $1.289 billion and $86 million USD respectively per year [14,19]. In Europe, the annual cost of foodborne illness was estimated to be $171 million USD in Sweden [6] and $2 million USD in Croatia [20]. Many estimates for specific foodborne pathogens, or groups of pathogens, have been published [4,21,22]. Although economic estimates for foodborne illness have not been completed in Canada in the past 20 years [23], it has been recently estimated that 4 million episodes of domestically acquired foodborne illness occur annually in Canada [24].

Research has indicated that COI studies employ varied methodological approaches, and that there is little consistency in the cost inventories used in the COI literature [7-10]. This is an issue when interpreting and designing new COI studies, and also when comparing existing estimates for the same illnesses. When designing a study, it is difficult to determine which types of costs to include (e.g., direct, indirect, societal), which component costs in those categories to include (e.g., treatment costs, productivity losses, industry costs), and the level of detail by which costs should be estimated (e.g., which types of treatment or industry costs to include). It is also challenging to interpret or compare estimates without fully understanding which component costs were included in a study. Another concern is the lack of transparency when describing how specific component costs were estimated and the data sources being used for such estimates [8,25,26].

To investigate the reporting of component costs in the cost of foodborne illness literature, along with their relevant data sources, a review of the evidence is needed. The scoping study, or scoping review, is one approach used to survey the literature and aims to map the key concepts underpinning a research area [27]. The framework for conducting a scoping review emphasizes that the methods used throughout all stages of the process are conducted in a rigorous and transparent way. The process should be documented in sufficient detail to enable the review to be replicated by others, and this explicit approach increases the reliability of the findings. Unlike a systematic review, a scoping study does not often lead to the statistical pooling of quantitative evidence from various studies, as is often done in meta-analyses of systematic review data. While a scoping study uses an analytical framework or thematic construction to present the evidence, there is no attempt made to present the weight or quality of evidence in relation to particular policies or interventions [27,28].

This study employed a scoping review methodology to address the research question: “What are the component costs and the respective data sources being used for estimating the cost of foodborne illnesses in a population?”

## Methods

### Scoping review methodology

The scoping review framework published by Arksey and O’Malley in 2005 [27] includes 5 required stages which were followed in the present study. Many other scoping reviews have subsequently used this framework as a guideline [29].

### Identifying the research question

The research team, consisting of academic and government researchers with expertise in the areas of foodborne illness and public health, jointly determined how to synthesize the cost of foodborne illness literature through a series of in-person meetings. The government researchers also contributed as potential end-users of the information obtained from a review in this area. The goals were to identify the different component costs that have been included when determining the cost of foodborne illnesses, and to identify the data sources used to calculate these estimates.

### Identifying relevant studies

Two comprehensive electronic databases were chosen for the literature search. The MEDLINE (PubMed) database was used to identify studies from the human medical literature whereas studies from the animal health literature were located in the AGRICOLA database. Prior to the searches, 15 studies were identified by the research team as being highly relevant to a review in this area. The identification of these studies following relevance screening was used to verify the comprehensiveness of the search. Broad keyword searches were performed between October 27th and November 1st 2012 to identify studies that addressed the COI of any infectious disease, including foodborne illnesses (Table 1). Search terms were selected by extensive review of the terminology used in the titles and abstracts of the 15 studies of known relevance. No restrictions were placed on date, country, or language of publication during the searches. A broad search approach (i.e., using infectious disease keywords) was used initially. This allowed for later refinement of the data extraction process to address the specific research question for the study reported herein. The ‘peer-reviewed’ filter was unchecked in the AGRICOLA search. All of the search results were imported into RefWorks Reference Management Software (ProQuest LLP, 2012), and duplicate citations were removed using the close and exact-match functions.

**Table 1:** **Scoping review keyword search strategy to identify cost-of-illness studies for infectious diseases**^**a**^

**Foodborne keywords**	**Communicable keywords**	**Cost keywords**
Foodborne illness	Communicable	Cost
Foodborne illnesses	Communicable disease	Costs
Food-borne illness	Communicable diseases	Cost-of-illness
Food-borne illnesses	Communicable illness	Cost of illness
Foodborne disease	Communicable illnesses	Cost-of-illnesses
Foodborne diseases	Infectious	Cost of illnesses
Food-borne disease	Infectious disease	Costs-of-illness
Food-borne diseases	Infectious diseases	Costs of illness
Foodborne infection	Infectious illness	Costs-of-illnesses
Foodborne infections	Infectious illnesses	Costs of illnesses
Food-borne infection	Transmissible	Coi
Food-borne infections	Transmissible disease	Direct Costs
Food poison	Transmissible diseases	Direct Cost
Food poisoning	Transmissible illness	Indirect Costs
Foodborne poison	Transmissible illnesses	Indirect Cost
Foodborne poisoning		Economic
Food-borne poison		Economics
Food-borne poisoning		Economy
Foodborne pathogen		Economical
Foodborne pathogens		Financial
Food-borne pathogen		Monetary
Food-borne pathogens		Money
O157		Expenditure
VTEC		Expenditures
STEC		Dollar-value
O157:H7		Dollar value
Salmonella		
Campylobacter		

^a^Keywords in each column (Foodborne, Communicable, Cost) were combined with the Boolean operator ‘OR’ and then each of the combined categories were further combined with the operator ‘AND’ in the database searches.

### Study selection

Prior to screening, reviewers were provided with instructional documents that outlined the objectives of the review and how the results would be presented (i.e., using empty shell tables). The 15 studies of known relevance were also provided to the reviewers. Subsequently, titles and abstracts of 250 test studies were independently screened by two reviewers, and also by a member of the research team. The 250 test studies were selected at random from those identified by the literature searches. Any disagreements during the testing stage were discussed by all 3 of the reviewers, and differences were resolved by consensus. Two levels of relevance screening were performed. Each level was based on reviews of the title and abstract only, with the second level of screening also serving as a categorization step. Both levels of screening were performed independently by two reviewers. The first round of screening, which included all citations from the database searches, identified studies that described the COI of any infectious (communicable) disease, including foodborne illnesses, while excluding cost-effectiveness studies for specific interventions. A standardized relevance screening tool was created in Microsoft Excel (Version 2007). A Cohen’s kappa coefficient was calculated to establish a minimum level of agreement between each reviewer following the first relevance screening round. If the level of agreement was found to be poor (i.e., raised concerns among the reviewers), a third reviewer would have been used and the first round of relevance screening would be repeated.

Studies selected after the first round of screening underwent a second level of screening, whereby each of the infectious and foodborne disease COI studies were further classified into those that described the cost of foodborne illnesses in humans, foodborne illnesses in animals, infectious diseases in humans, infectious diseases in animals, a combination of any of these categories, none of these categories (the study did not describe the cost of an infectious or foodborne illness), and as studies in which relevance could not be determined using the title and abstract. Following the second level of relevance screening, the study focused on the COI of foodborne diseases in humans only, and all other categories of studies were excluded. Results from each level of screening were compared between reviewers and conflicts resolved by consensus through an open discussion.

### Charting the data

Citations describing the cost of foodborne illnesses in humans (with or without other infectious illnesses) and citations where relevance could not be determined using the title and abstract were retrieved in full text. A standardized data-charting form was created in Microsoft Excel (Version 2007). Training for data extraction was performed using instruction forms and 7 full text studies. Data extraction was conducted by two independent researchers, and the completed forms were compared for comprehensiveness. Therefore, if one researcher extracted data that the other had omitted, the study was re-examined by both reviewers, and differences in extracted data were resolved by consensus.

The data-charting form had two sections, the first for gathering descriptive information on the relevant studies (Table 2) and the second for gathering the data of interest: the individual and societal level component costs included in the studies and the data sources for those estimations (Tables 3 and 4). Descriptive data included information on the title of the study, whether it was available in English, whether it directly estimated COI due to one or more foodborne pathogens, and whether it described the component costs for the estimate. The year of publication, country of publication, and a list of foodborne pathogens included in the study were also collected. All component cost data were extracted at the level of detail reported in each study rather than using pre-determined categories for data extraction. Therefore, the specificity and detail in the extracted data were representative of the level of detail reported in the paper. The source of data for each component cost was also collected, detailing whether the data for the estimation came directly from a: survey, pre-existing databases, hospital records, an online calculator (e.g., Economic Research Service of the United States Department of Agriculture’s foodborne illness cost calculator) [30], the literature, population statistics, census data, outbreak data, or expert opinion.

**Table 2:** Descriptive information of the 84 cost of foodborne illness studies published between 1972 and 2012 identified from a scoping review

**Study type**		**No. of studies**
	Cost-of-illness	74
	Not cost-of-illness (described component costs)	10
	Non-English (Excluded)^a^	10
	Non-relevant studies (Excluded)^b^	14
**Region of publication**		
	North America	43
	Europe	29
	Asia	6
	Oceania	5
	Africa	1
	South America	0
**Year of publication**		
	2002 - 2012	36
	1992 - 2001	31
	1982 - 1991	15
	1972 - 1981	2
	Before 1972	0
**Foodborne pathogens**		**No. included in studies**
	**1.** Non-typhoidal *Salmonella* spp.	51
	**2.** Shiga-toxin producing *Escherichia coli*	34
	**3.***Campylobacter* spp.	27
	**4.***Vibrio* spp.	19
	**5.***Staphylococcus aureus*	17
	**6.***Listeria monocytogenes*	16
	**7.***Clostridium perfringens*	12
	**8.***Salmonella typhi*	12
	**9.***Clostridium botulinum*	11
	**10.***Shigella spp.*	10
Protozoa and parasites^c^		29
Viruses^d^		28
Other bacteria^e^		24

^a^Non-English study languages: Swedish (4), German (3), Italian (1), Danish (1), Russian (1).

^b^Non-relevant studies: Did not describe component costs (i.e., studies that were identified as relevant through both levels of screening, but did not provide the data of interest).

^c^*Toxoplasma gondii* (10), *Cyptosporidium* spp. (7), *Cyclospora cayetanensis* (5), *Trichinella* spp. (3), *Giardia lamblia* (3), *Taenia* spp. (1).

^d^Norovirus (9), Hepatitis A (6), Rotavirus (6), Astrovirus (4), Saprovirus (2), Adenovirus (1).

^e^*Yersinia enterocolitica* (8), *Bacillus cereus* (6), *Brucella* spp. (4), *Streptococcus* spp. (4), *Mycobacterium bovis* (1), *Plesiomonas* spp. (1).

**Table 3:** Individual level component costs and data sources from 84 cost of foodborne illness studies published between 1972 and 2012 identified from a scoping review

**Component costs**	**N**^**a**^	**Data sources**^**b**^											
**Direct costs**		**H**	**D**	**L**	**OC**	**S**	**O**	**C**	**P**	**E**	**M**	**N/P**	**N/A**
**Medical costs**	**16**	1	3	2	-	-	3	-	-	-	-	2	5
**Treatment costs**	**14**	4	2	3	-	1	-	-	-	-	3	-	1
Drug costs	**29**	3	3	6	1	3	2	1	-	-	4	3	3
Prescription	**18**	3	3	1	-	6	-	1	-	-	1	1	2
Over-the-counter	**6**	-	1	1	-	4	-	-	-	-	-	-	-
Non-personal transportation (ambulance)	**12**	1	1	3	1	-	-	-	1	2	1	-	2
Rehabilitation	**10**	-	1	3	1	-	1	1	-	-	2	-	1
Materials (disposable/non-disposable)	**7**	1	2	-	-	1	1	1	-	-	-	1	-
Home visits	**5**	-	-	-	-	-	-	-	-	-	1	3	1
Rehydration treatment	**2**	1	-	-	-	1	-	-	-	-	-	-	-
Palliative care	**1**	-	1	-	-	-	-	-	-	-	-	-	-
**Laboratory costs**	**20**	5	3	2	1	2	1	-	-	1	2	1	2
Pathogen diagnosis and analysis	**15**	3	2	1	-	2	3	-	-	-	3	-	1
Ancillary diagnostics	**10**	3	2	1	1	-	2	-	-	-	-	-	1
Laboratory sampling	**7**	1	1	2	-	3	-	-	-	-	-	-	-
**Personnel costs**	**7**	2	3	-	-	2	-	-	-	-	-	-	-
Physician	**31**	1	6	5	1	3	3	1	-	1	6	2	2
General practitioner physician	**17**	2	5	4	-	3	-	-	-	-	3	-	-
Non-physician	**2**	-	-	-	-	1	-	-	-	-	1	-	-
Nurses	**4**	1	-	1	-	-	1	-	-	-	1	-	-
Laboratory technician	**3**	1	-	-	-	-	2	-	-	-	-	-	-
Consultants	**7**	-	1	2	-	1	2	-	-	-	1	-	-
Specialists	**5**	1	1	-	-	-	-	-	-	-	1	1	1
**Hospital services costs**	**46**	5	6	11	1	2	5	1	-	1	7	3	4
Emergency room	**14**	4	3	1	-	1	-	-	-	-	3	-	2
Intensive care unit	**5**	1	1	-	-	-	-	1	-	-	2	-	-
Surgical services	**3**	-	1	-	-	-	-	-	-	-	1	-	1
Dialysis	**2**	1	1	-	-	-	-	-	-	-	-	-	-
**Community services (out-patient) costs**	**11**	-	3	1	-	-	-	-	-	-	3	1	3
**Long-term care services costs**	**4**	-	2	-	-	-	-	-	-	-	-	1	1
**Indirect costs**													
**Productivity losses**	**30**	-	2	6	1	4	2	-	-	-	3	4	8
Due to sick leave from work (patient)	**42**	1	6	7	1	10	3	1	1	-	4	1	6
Due to caring for others (caregiver)	**19**	-	5	3	-	6	2	-	-	-	3	-	-
Due to care of sick children	**11**	-	2	1	1	1	1	-	-	-	1	2	2
Lost leisure time	**14**	-	3	6	1	-	1	-	-	-	1	-	2
Due to long-term or permanent disability	**8**	-	1	2	-	-	2	-	-	-	2	-	1
**Patient transportation (non-ambulance) costs**	**20**	-	2	6	-	5	1	-	1	-	1	2	2
For visitors and relatives	**6**	-	-	1	-	1	1	-	-	-	1	-	2
Parking fees	**1**	-	-	-	-	1	-	-	-	-	-	-	-
**Additional costs**	-	-	-	-	-	-	-	-	-	-	-	-	-
Value-of-life lost	**29**	-	1	15	1	1	1	-	-	-	3	2	5
Pain and suffering	**21**	-	-	8	2	2	1	-	-	-	2	2	4
Risk aversion behaviours	**9**	2	2	-	1	2	-	-	-	-	-	-	2
Facility (operational) costs	**9**	1	2	2	1	-	-	-	-	-	2	1	-
Non-medical materials	**7**	-	-	1	1	3	-	-	-	-	-	1	1
**Totals**		**49**	**84**	**108**	**17**	**72**	**41**	**8**	**3**	**5**	**69**	**34**	**68**

^a^Counts represent the number of studies reporting each component cost category at the specified level of detail. Therefore, a study that is counted as including a higher-level category (e.g., treatment costs) cannot contribute a count to a lower-level category (e.g., drug costs) within the same grouping, and vice versa.

^b^D: Database, L: Literature, OC: Online calculator S: Survey, O: Outbreak data, C: Census, P: Population statistics, E: Expert opinion, M: Multiple, N/P: Not provided, N/A: Not applicable.

**Table 4:** Societal level component costs and data sources from 84 cost of foodborne illness studies published between 1972 and 2012 identified from a scoping review

**Component costs**	**N**^**a**^	**Data sources**^**b**^										
		**D**	**L**	**OC**	**S**	**O**	**C**	**P**	**E**	**M**	**N/P**	**N/A**
**Industry costs**	**10**	3	3	1	1	-	-	-	-	-	-	2
Losses to businesses	**13**	1	2	-	1	1	-	-	-	2	2	4
Reduced product demand	**7**	-	3	-	1	-	-	-	-	1	-	2
Advertising to regain customer trust	**6**	1	2	1	-	-	-	-	-	-	-	2
Losses to food service establishments	**5**	-	2	-	-	1	-	-	-	1	-	1
Product spoilage	**4**	-	1	-	-	1	-	-	-	-	-	2
Product recall	**11**	-	3	1	2	-	-	1	-	1	-	3
Farm-related costs	**3**	-	2	-	-	-	-	-	-	-	-	1
Herd slaughter	**4**	-	1	1	-	-	-	-	1	-	-	1
Farmers compensation	**2**	2	-	-	-	-	-	-	-	-	-	-
Increased time to market	**2**	-	1	1	-	-	-	-	-	-	-	-
Adjusted manufacturing procedures	**9**	-	3	-	2	1	-	-	-	1	-	2
Plant closure and bankruptcy	**6**	-	2	-	1	1	-	-	-	1	-	1
Equipment	**5**	1	2	-	-	-	-	-	1	1	-	-
**Public health costs**	**5**	2	-	-	1	-	-	-	-	-	-	2
Outbreak investigation costs	**15**	-	5	-	4	2	-	-	-	-	-	4
Laboratory testing	**10**	1	3	-	1	2	-	-	-	1	-	2
Personnel	**7**	-	-	-	1	4	-	-	-	1	-	1
On-site treatment	**3**	-	2	-	-	-	-	-	-	-	-	1
Clean up (including food destruction)	**3**	-	3	-	-	-	-	-	-	-	-	-
Consumables	**3**	-	-	-	1	2	-	-	-	-	-	-
Administration	**2**	1	-	-	-	1	-	-	-	-	-	-
Source identification	**2**	-	-	-	1	1	-	-	-	-	-	-
Make-shift food services	**2**	-	-	-	-	2	-	-	-	-	-	-
Travel	**2**	-	-	-	-	1	-	1	-	-	-	-
Prevention	**6**	2	-	-	-	-	-	-	1	-	-	3
Surveillance (including database creation)	**11**	2	5	1	1	-	-	-	-	1	-	1
Educational campaigns	**7**	-	2	1	-	-	-	-	-	1	2	1
Research	**6**	2	1	-	-	1	-	-	-	1	-	1
Vaccination programs	**2**	-	1	-	-	-	-	-	-	-	-	1
**Legal costs**	**6**	1	1	-	1	1	-	-	-	1	-	1
Product liability suits	**5**	-	2	-	1	1	-	-	-	-	-	1
Insurance-related	**4**	-	2	-	-	1	-	-	-	1	-	-
Victim (individual) settlements	**3**	-	-	-	-	-	-	-	-	-	-	3
Class action (group) settlements	**1**	-	-	-	-	-	-	-	-	-	-	1
Out-of-court settlements	**1**	-	-	-	-	-	-	-	-	1	-	-
Prosecution costs from public funds	**1**	-	-	-	-	-	-	-	-	-	-	1
Jail sentences	**1**	-	-	-	-	-	-	-	-	1	-	-
**Government and regulatory costs**	**7**	2	-	-	1	1	-	-	1	1	-	1
Regulatory fines and enforcement	**6**	-	2	1	-	-	-	1	-	1	-	1
Local authority investigations	**5**	1	1	-	2	-	-	-	-	-	-	1
Public inquiry	**1**	-	-	-	-	-	-	1	-	-	-	-
Policy implementation and monitoring	**1**	-	-	-	-	-	-	-	-	-	-	1
**Totals**		**22**	**57**	**8**	**23**	**25**	**0**	**4**	**4**	**19**	**4**	**49**

^a^Counts represent the number of studies reporting each component cost category at the specified level of detail. Therefore, a study that is counted as including a higher-level category (e.g., industry costs) cannot contribute a count to a lower-level category (e.g., product recall) within the same grouping, and vice versa.

^b^D: Database, L: Literature, OC: Online calculator S: Survey, O: Outbreak data, C: Census, P: Population statistics, E: Expert opinion, M: Multiple, N/P: Not provided, N/A: Not applicable.

### Collating, summarizing, and reporting the results

The primary goal of this step was to refine the information extracted from the studies into manageable groupings, or themes. Two researchers independently grouped the component cost and source data extracted from each paper into themes and differences were resolved by consensus throughout the process. The grouped information was summarized in categories of individual level (direct and indirect) and societal level component costs.

Tables 3 and 4 display the categories of direct and indirect costs included at the individual level and the data sources used for each component cost category. The categories were created based on the level of detail provided in the study and thus, some categories represent more detailed sub-categories. Therefore, a study which included ‘medical costs’ did not explicitly describe any other direct costs included in their COI estimate. Similarly, all of the studies that were categorized as including a broad component cost category (e.g., treatment costs, personnel costs, hospital service costs) were not counted towards including a more specific component in those categories. However, studies may have ultimately included these more specific costs in their estimates, but the components were unknown due to a superficial level of reporting detail.

Data sources were grouped as follows: if the authors of a study stated that the literature was used for an estimate, the source of data was described as ‘literature’. The original source of data in the cited literature may have been one of the other categories (e.g., a survey or pre-existing databases), however, the cited literature was not obtained to determine the actual data source. The same principle applied for databases, population statistics, outbreak data, or census data that may have been created using information from other data sources. This resulted in potential overlapping between data source categories, as only the immediate source of data was identified in the present study. Cost calculators often provide component cost estimates that have been amalgamated from a range of data sources, and are tools that can be used when estimating costs [31]. Sources of component cost data could also be described as not provided (N/P), not applicable (N/A), or as ‘multiple’ , meaning numerous component costs and data sources were described without specifying which data sources were used for a particular component cost estimate.

## Results

**Figure 1:**
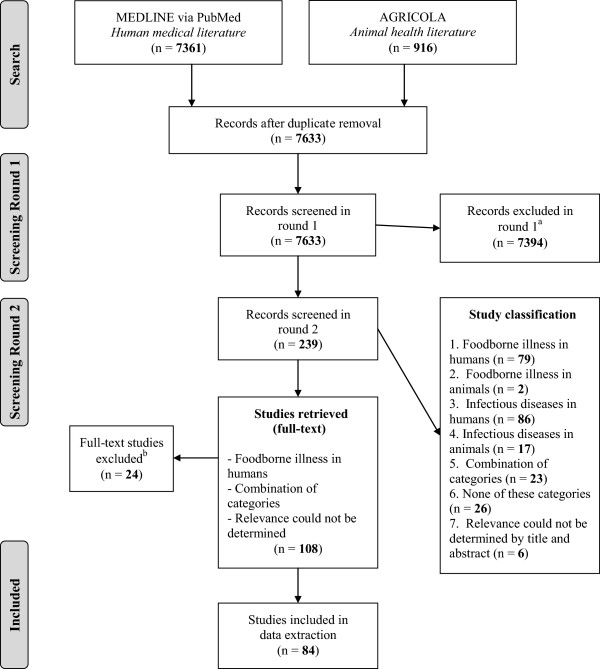
**Scoping review flow chart. **^a^Studies were excluded as they did not describe the COI of any infectious or foodborne illnesses. ^b^Ten non-English references were excluded (4 Swedish, 3 German, 1 Italian, 1 Danish, 1 Russian), as were an additional 14 studies that did not provide any information on component costs.

Following duplicate removal, the MEDLINE (PubMed) and AGRICOLA database searches yielded 7633 references to be screened for relevance (Figure 1). Of these, 7394 were excluded as they did not describe the COI of any infectious or foodborne illnesses. All of the 15 studies identified by the research team as being highly relevant to a review in this area prior to the literature searches were identified by the employed search strategy. The Cohen’s kappa coefficient was 0.89 for the first relevance screening round, indicating substantial agreement between the two reviewers [32]. In the second round of screening, the remaining 239 references were classified into 1 of 7 categories based on the type of COI that was estimated (e.g., cost of foodborne illness in humans). Classifications for studies that fell into multiple categories, none of the categories, or studies where the relevance could not be determined were also used.

Following the second round of relevance screening, references that focused on foodborne illness in humans, a combination of categories, and those where relevance could not be determined from the title and abstract were selected (n = 108). Ten non-English references were identified and excluded, as were an additional 14 studies that did not provide any information on component costs. Therefore, 84 studies ultimately underwent data extraction. These studies described studies that directly estimated the cost of foodborne illnesses and studies that described component costs but did not provide an estimate.

The majority of the studies (n = 74, 88%) calculated the cost of a foodborne illness (or a group of foodborne illnesses) and described the component costs included in the estimates (Table 2). Ten studies described component costs, but did not directly calculate the cost of a foodborne illness. Papers in this latter category were foodborne illness prioritization studies, burden of foodborne illness reviews, and conceptual studies such as cost of foodborne illness frameworks. Although the objective of this group of studies was not to calculate the cost of a foodborne illness, they did describe component costs and were therefore included. Data source identification was not applicable (N/A) for these ten studies. Of the 74 COI studies, 36 (49%) estimated the cost of a single foodborne pathogen while 38 (51%) examined multiple pathogens. Among all included studies (n = 84), most (80%) were published in the last two decades (1992–2012) in North America (51%) and Europe (35%). The 10 most frequently estimated costs were those due to illnesses caused by bacterial foodborne pathogens, with non-typhoidal *Salmonella* spp. (n = 51, i.e., COI for this pathogen was reported in 51 of 74 studies estimating costs), shiga-toxin producing *E. coli* (n = 34), and *Campylobacter* spp. (n = 27) being the most commonly studied. Additional bacterial foodborne pathogens were included in multiple studies (refer to footnotes of Table 2), as well as foodborne viruses, protozoa, and parasites.

Among the 84 studies included in the review, 40 (48%) studies described both individual (direct and indirect) and societal level costs. Twenty-seven (32%) studies described individual level costs only, while 10 (12%) studies described direct costs exclusively. Three studies solely examined the societal costs associated with foodborne illness. The remaining studies described both societal and direct individual costs (n = 2) or societal and indirect individual costs (n = 2).

The direct individual level component costs most often included were broadly described as hospital services costs (n = 46) without explicitly describing which hospital service costs they estimated (e.g., emergency room costs, intensive care costs, surgical services costs, dialysis costs). Physician costs, a component of personnel costs, were commonly included (n = 31) along with drug costs (n = 29), a component of treatment costs. Other studies described these and other costs at a greater depth (e.g., prescription and over-the-counter costs as a component of drug costs), and there was substantial variation in the specificity and detail among studies when describing component costs. The most commonly reported indirect component cost was productivity losses due to sick leave from work (n = 42). A large number of studies included a cost estimate for ‘productivity losses’ without specifically stating which individual was experiencing the loss of output (e.g., the patient or caregiver) (n = 30). The value-of-life lost was estimated in 29 studies while costs broadly described as ‘personal transportation’ expenses were calculated in 20 studies.

Prior estimates published in the literature were the most commonly used source of individual level component cost data (used 108 times), followed by databases (84 times) and surveys (72 times). Multiple sources were listed for component cost estimates on 69 occasions. These studies included a description of multiple component costs and data sources without specifying which data sources were used for a particular component cost estimate. No data sources were provided for component cost estimates 34 times, and data sources were not applicable for 68 of the component costs. These component costs came from the 10 studies that did not directly estimate a cost of foodborne illness.

The societal level component costs that were most often included in cost of foodborne illness studies were outbreak investigation costs (a component of public health costs, n = 15), losses incurred by businesses (a component of industry cost, n = 13), costs associated with product recall (a component of industry costs, n = 11), and costs related to public health surveillance of foodborne illness (n = 11) (Table 4). Other societal costs included in some studies were legal costs and government related (regulatory) costs.

Similar to the individual costs, prior estimates published in the literature were the most commonly used source of component cost data for societal costs (used 57 times). Outbreak data, surveys, and pre-existing databases were used 25, 23, and 22 times respectively. Multiple sources were listed for 19 component costs. These studies described numerous component costs and data sources without specifying which data sources were used for a particular component cost estimate. No data sources were provided for component cost estimates 4 times, and data sources were not applicable on 49 occasions.

## Discussion

This scoping review explored component costs of foodborne illness and sources of data for the cost estimates. High variability in terms of the depth and breadth of individual and societal level cost components and the data sources being used in the published cost of foodborne illness literature was observed. Rather than being guided by a highly specific research question and particular study designs, a scoping review is guided by the broad requirement of identifying all relevant literature that pertains to the research question [27]. Due to the potential usefulness of COI studies to inform decision makers, it is important that COI estimates are derived in a uniform, consistent, and transparent manner [12,25,26]. To address the issues of uniformity in cost inventories and transparency in data source usage, a better understanding of which cost components are included and how they are described in the published cost of foodborne illness literature is critical.

Most studies were based in North America and Europe, indicating that the results are more applicable to developed country contexts and may not represent foodborne illness component costs and data sources in developing nations. This may be due to a lack of resources to conduct COI studies or it may be a reflection of other infectious disease priorities for developing countries. The majority of the cost of foodborne illness studies identified have been published in the past two decades (1992–2012), which is a trend observed in all COI literature [14]. The 10 most frequently estimated costs were those due to illnesses caused by bacterial foodborne pathogens. This was expected as these pathogens are cited as carrying a large burden in terms of the number of illnesses, hospitalizations, and deaths [17].

The primary results from this study are the reporting patterns of component costs in the cost of foodborne illness literature along with the sources of data for each estimate. In regards to the breadth of cost inventories, almost half of the studies (48%) included individual level costs (direct and indirect) and societal level costs in their estimates. This indicated that many studies are estimating a wide spectrum of costs associated with foodborne illnesses. Fewer studies included societal costs compared to individual costs. Societal level costs may be more difficult to calculate, as attributing costs incurred at the population level due to a particular illness might be more challenging than estimating a direct or indirect cost associated with an individual person [33]. Additionally, societal costs may not be applicable given a study’s perspective (e.g., a study estimating healthcare-related costs may omit societal costs). In the 84 studies included in the review, there was a high level of variability in the reporting detail of individual and societal level component costs. For instance, 16 studies broadly included ‘medical costs’ in their estimates as the only individual direct cost, while the remainder of studies estimating direct costs included more specific components of medical costs such as treatment costs, laboratory costs, personnel costs, hospital service costs, community services costs, and long-term care services costs. Numerous papers provided even greater detail, with studies delineating particular components in these broader categories. For example, specific treatment-related components such as drug and rehabilitation costs were described in certain studies. The variability in reporting detail can also be seen in the indirect individual and societal level costs, which indicates that although a greater level of specificity can be achieved when calculating component costs, certain studies elect to estimate costs more superficially. This is an issue because it does not allow the end-user of a COI study to fully understand which types of costs were included in an overall estimation. In turn, this makes the economic burden of an illness more difficult to interpret and understand and reduces the feasibility of meaningfully comparing two studies for the same disease.

The component costs presented in Tables 3 and 4 were aggregated from all of the relevant cost of foodborne illness studies identified during the review. However, certain costs may only be relevant for a particular pathogen or chronic sequelae. An example of this would be intensive care unit (ICU) costs incurred due to shiga-toxin producing *E. coli* infections, which may not be as relevant to other foodborne illnesses. A future study could determine which costs are pathogen-specific and which are commonly included across all foodborne illnesses. Additionally, of the 74 COI studies, 36 estimated the cost of a single foodborne pathogen while 38 examined multiple pathogens. Further research could determine if the component cost inclusion and reporting detail differs in single-versus multiple-pathogen studies, and to explore the implications of this factor when comparing or combining COI estimates.

A further consideration is the impact of data sources on a cost of foodborne illness estimate. A wide variety of data sources were used to estimate component costs of foodborne illness. Certain data sources may be more credible than others. For example, it could be argued that costs estimated by expert opinion are more subjective than estimates taken from hospital records. Future research could compare specific component cost estimates for a particular pathogen using varying data sources to determine the impact of using different sources of data. However, because there was an overlapping of data sources (e.g., an estimate taken from the literature may have come from a survey), data source variability may be less substantial than it appears, as only the immediate source of data was identified in the present study. Additionally, when a study reports a data source (e.g., the literature) without identifying the origin of the information, which may in fact be another data category (e.g., a survey, hospital records, pre-existing database), it does not allow the reader to easily evaluate the appropriateness or validity of the data source for the estimate. Also of concern is the number of component cost estimates that could not be linked to a particular source of data. For both individual and societal costs, multiple sources were listed for 88 component costs across 14 different studies. These studies described numerous component costs and data sources without specifying which data sources were used for a particular component cost estimate. Data sources were not provided for 38 individual and societal level component costs, meaning that an estimate was included without any explanation of where it came from or how it was deduced. These are issues of inadequate reporting that inhibits repeatability of these estimates.

Proponents of COI research have cited that one of the major strengths of these studies is the potential to compare one estimate to another [12,25]. In an era where evidence-informed decision making is at the forefront, synthesizing the evidence from high quality studies is an important step in making an informed decision [34]. Numerous studies dating back to 1982 have stressed that researchers should standardize their COI methodologies to improve the consistency and comparability of estimates [12,13,26]. These studies claim that if two otherwise comparable studies have included different components when estimating a cost of an illness, it would not be meaningful to compare them. If researchers continue publishing cost of foodborne illness studies while using different cost inventories (i.e., studies which contain a wide range of component costs reported with varying levels of detail), this trend of insular estimates with limited comparability will continue. Therefore, the research community engaging in COI studies may benefit from a discussion of minimum criteria for component cost and data source reporting. This scoping review illustrates the breadth of published cost inventories in the cost of foodborne illness literature and the depth to which they have been reported. By using this scoping review as evidence that there is a lack of standardization in cost inventories in the cost of foodborne illness literature, and to promote greater transparency and detail of data source reporting, there will be an increase in cost of foodborne illness research that can be interpreted and compared in a meaningful way.

During the literature search, a formal search of the grey (unpublished) literature was not conducted. However, the peer-reviewed filter was left unchecked during the AGRICOLA database search and relevant grey literature identified at this stage was included in the review. Also, by only searching a single animal health-related database (i.e., AGRICOLA), the number of studies identified as describing the cost of foodborne and infectious illnesses in animals may be an underestimation. However, we do not believe that this has biased the results, as the study reported herein focused on costs related to foodborne illnesses in humans only. Non-English language papers were excluded from the present study, and therefore, these results may only be applicable to English speaking countries. Lastly, an optional stage (step 6) in the Arksey and O’Malley [27] scoping review framework is to involve potential end-users in the scoping review process. Although a consultation was not conducted per se, members of the research team, who are also end-users of the information, were directly involved in the identification of the research question and in outlining the goals of the review.

## Conclusions

Individual and societal level costs have been included in a substantial number of foodborne illness cost estimates, indicating that many studies are using cost inventories that estimate a variety of types of costs. The depth and breadth of individual and societal level cost inventories in the cost of foodborne illness literature were highly variable. This scoping review can be used as evidence that there is a lack of standardization in cost inventories in the cost of foodborne illness literature, and to promote greater transparency and detail of data source reporting. It should be noted that certain costs may be pathogen specific and the results of the present study should be interpreted with that in mind. Lastly, the results illustrate that there are a wide variety of data sources available to estimate component costs of foodborne illness. Efforts should be made to select credible and current sources when determining the costs associated with foodborne illness, and to report the specific source of data for each component cost estimate. These suggestions will help address the issues of uniformity in component cost selection and reporting. By conforming to a more standardized cost inventory for cost of foodborne illness studies, and by reporting data sources in more detail, there will be an increase in cost of foodborne illness research that can be interpreted and compared in a meaningful way.

## Competing interests

The authors declare that they have no competing interests.

## Authors’ contributions

TM: Is a former graduate student at the University of Guelph and a PhD student in Epidemiology at McGill University. TM participated in the design of the study, carried out the scoping review methodology, interpreted the results, and drafted the manuscript. JMS: Is the Director of the Centre for Public Health and Zoonoses and a Professor in the Department of Population Medicine (University of Guelph). JMS participated in the design of the study, assisted in carrying out the scoping review, and assisted with data interpretation. MKT (Epidemiologist, Public Health Agency of Canada), AP (Associate Professor in the Department of Population Medicine, University of Guelph), and AF (Chief in the Risk Integration Synthesis and Knowledge Section, Public Health Agency of Canada) participated in the design of the study and provided feedback on the manuscript. All authors read and approved the final manuscript.

## Pre-publication history

The pre-publication history for this paper can be accessed here:

http://www.biomedcentral.com/1471-2458/14/509/prepub
